# Knockdown of heat shock transcription factor 1 decreases temperature stress tolerance in *Bemisia tabaci* MED

**DOI:** 10.1038/s41598-022-19788-z

**Published:** 2022-09-26

**Authors:** Jing Bai, Yun-Cai Liu, Ran Wei, Yu-Cheng Wang, Wei-Rong Gong, Yu-Zhou Du

**Affiliations:** 1 College of Horticulture and Plant Protection and Institute of Applied Entomology, Yangzhou University, Yangzhou, 225009 China; 2 Plant Protection and Quarantine Station of Jiangsu Province, Nanjing, 21003 China; 3 Joint International Research Laboratory of Agriculture and Agri-Product Safety, the Ministry of Education, Yangzhou University, Yangzhou, China

**Keywords:** Gene expression analysis, RNAi

## Abstract

The primary function of heat shock transcription factor (HSF) in the heat shock response is to activate the transcription of genes encoding heat shock proteins (HSPs). The phloem-feeding insect *Bemisia tabaci* (Gennadius) is an important pest of cotton, vegetables and ornamentals that transmits several plant viruses and causes enormous agricultural losses. In this study, the gene encoding HSF (*Bthsf1*) was characterized in MED *B. tabaci*. The full-length cDNA encoded a protein of 652 amino acids with an isoelectric point of 5.55. The *Bt*HSF1 deduced amino acid sequence showed strong similarity to HSF in other insects. Expression analyses using quantitative real-time PCR indicated that *Bthsf1* was significantly up-regulated in *B. tabaci* adults and pupae during thermal stress. Although *Bthsf1* was induced by both hot and cold stress, the amplitude of expression was greater in the former. *Bthsf1* had distinct, significant differences in expression pattern during different duration of high but not low temperature stress. Oral ingestion of *dsBthsf1* repressed the expression of *Bthsf1* and four heat shock proteins (*Bthsp90*, *Bthsp70-3*, *Bthsp20* and *Bthsp19.5*) in MED *B. tabaci* during hot and cold stress. In conclusion, our results show that *Bthsf1* is differentially expressed during high and low temperature stress and regulates the transcription of multiple *hsps* in MED *B. tabaci*.

## Introduction

Insects are continually stressed by various environmental factors, and thermal stress is perhaps the most common and direct of these stressors. In response to thermal stress, insects deploy innate resistance mechanisms to alleviate the damage caused by temperature stress^1–3^. Among these, heat shock proteins (HSPs) directly respond to temperature stress and have a pivotal role in protecting insects from thermal damage^4^. In insects, HSPs can be subdivided into HSP100, HSP90, HSP70, HSP60, HSP40 and small heat shock proteins (sHSPs) depending on their structure, function and molecular weight^5–8^. Studies have shown that HSPs interact with heat shock elements (HSE) in the promoter region of genes via heat shock transcription factors (HSFs); this interaction facilitates the recruitment of other transcription factors and the formation of a transcription complex that promotes *hsp* expression^9,10^.

Heat shock transcription factors are crucial regulatory factors of the heat shock response that are conserved in eukaryotes^10,11^. HSFs are commonly divided into four types, including HSF1, HSF2, HSF3 and HSF4; of these HSF1 is considered to be the main regulator of *hsp* expression^10,12^. HSF1 is highly conserved in *Drosophila melanogaster*, yeast and vertebrates, and its function cannot be replaced by the other three HSF regulators. HSF1 is expressed in response to heat stress in most tissues and cells^13^ and has conserved domains: DNA-binding domain (DBD)^14^. After DNA binding, oligomerization, and nuclear localization, HSF1 regulates the expression of stress-induced *hsps* to foster the organismal response to environmental stressors such as high temperature, heavy metals, and protease inhibitors^15^. The function of HSF1 has been well-studied in insects^16,17^. In *Drosophila*, *hsf* is constitutively expressed in the cytoplasm and nucleus. In vitro studies have confirmed that *Drosophila* HSF can directly respond to high temperature and oxidative stress, thus indicating that HSF acts as an "thermometer" to regulate the stability of the intracellular environment when physiological tolerance is exceeded^18^. In addition, a few reports exist documenting HSF1 in other insect species including *Helicoverpa armigera*, *Bombyx mori* and *Mamestra brassicae* and explained the role of HSF1 in the process of resistance to the external environmental temperature stress^19–21^.

The whitefly, *Bemisia tabaci* (Gennadius), is a species complex that contains 44 cryptic species^22^. It is polyphagous and colonizes over 600 known host plants^23,24^. *B. tabaci* feeds directly on plants, secretes honeydew, and disseminates plant viruses; it is an invasive pest that causes damage to host plants and serious economic losses to crop production worldwide^25,26^. The invasive species represented by MED cryptic species (*B. tabaci* Q) is the most serious form of this pest. It can spread rapidly and competes to replace indigenous species, including the MEAM1 cryptic species (*B. tabaci* B). The adaptability of the MED cryptic species is related to many external factors, including pesticide sensitivity, behavioral interactions and host range^27–31^. The malleability of the MED cryptic species is the primary reason it can quickly adapt to different habitats, including those with temperature extremes^32–34^.

Several studies have demonstrated that thermotolerance of the MED cryptic species correlates with *hsp* expression, especially *hsp90*, *hsp70* and *shsps*^33,35,36^. However, the relationship between these three *hsp* gene families and the whitefly heat shock transcription factor is not clear. In the present study, we cloned and identified the full-length gene encoding *B. tabaci* heat shock transcription factor 1 (*Bthsf1*) and analyzed its expression during temperature stress. RNA interference (RNAi) was used to further understand the role of *Bt*HSF1 in the regulation of *hsps* in *B. tabaci*, which may ultimately lead to improved control methods for this important pest.

## Results

### Sequence analysis of *Bthsf1*

**Figure 1:**
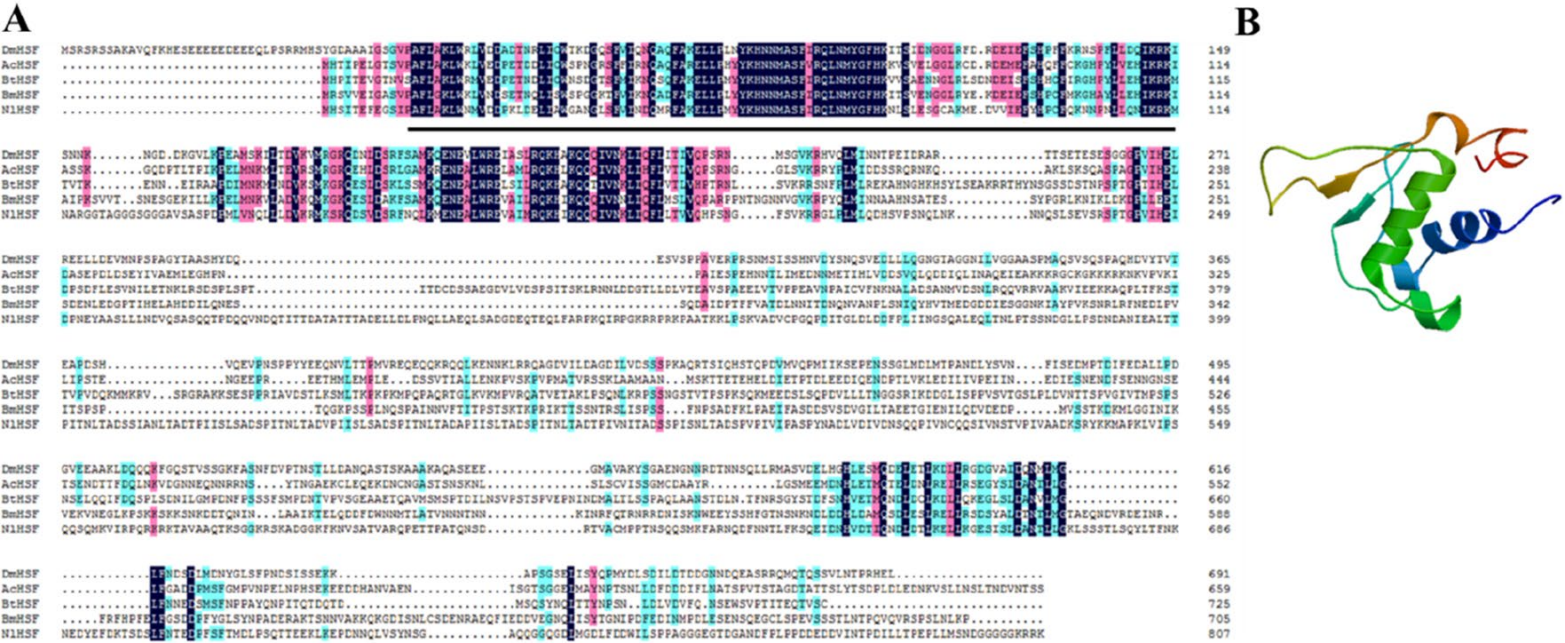
Multiple sequence alignment of HSF1 from various insect species and Structure of HSF1. (**A**) Alignment of the deduced amino acid sequences of *Bthsf1*, *Dmhsf*, *Achsf*, *Bmhsf* and *Nlhsf*. Abbreviations: *Bt*, *Bemisia tabaci*; *Dm*, *Drosophila melanogaster*; *Ac*, *Apis cerana*; *Bm*, *Bombyx mori*; *Nl*, *Nilaparvata lugens*. The DNA-binding (DBD) motif are underscored in black. Accession numbers of species are noted in Table S1. (**B**) 3D predicted structure of HSF1.

The full-length cDNA of *Bthsf1* was 2500 bp and encoded a predicted protein containing 725 amino acids (GenBank accession no. MW478139) (Fig. S1). The predicted protein product of *Bthsf1* was 80.23 kDa with an isoelectric point of 5.90. When the GenBank and PROSITE databases were compared, the *Bt*HSF1 deduced protein showed high similarity to the HSF1 family; InterPro analysis indicated that *Bt*HSF1 contained a conserved DNA-binding domain (DBD) at amino acid residues 10–114 (Fig. 1A). The 3D structure of HSF1 in *B. tabaci* was modeled using the DBD domain in *D. melanogaster* (SMTL ID: 1hkt.1) as a template (Fig. 1B); HSF1 showed 69.81% sequence identity to the *D. melanogaster* orthologue.

### Phylogenetic analysis of *Bt*HSF1

**Figure 2:**
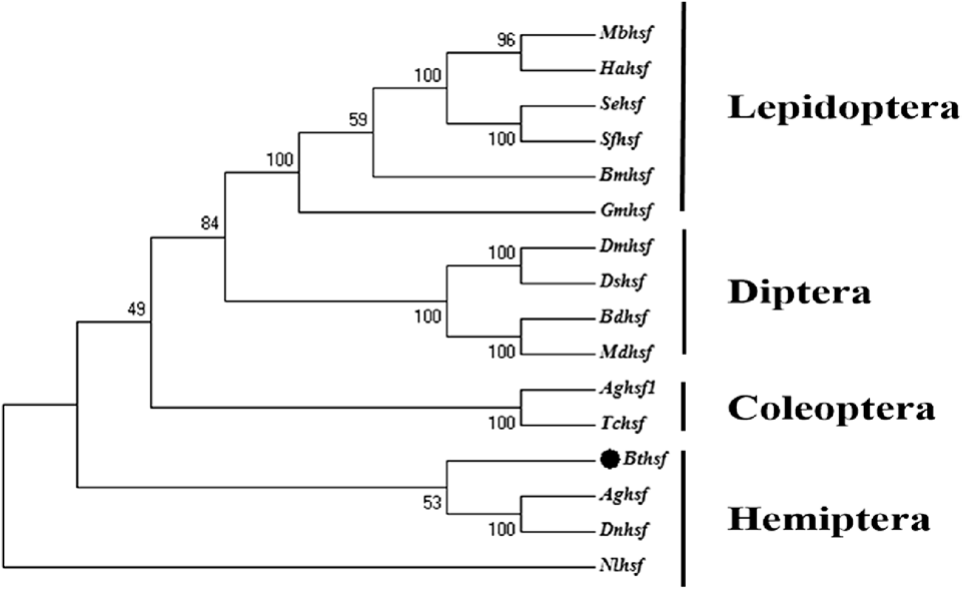
Phylogenetic analysis of HSF1 in *B. tabaci* and other insect species. Numbers on the branches are bootstrap values obtained from 1000 replicates. Accession numbers and abbreviations for the insect species are listed in Table S1.

The *Bt*HSF1 deduced amino acid sequence was compared with orthologous proteins in other insects. *B. tabaci* HSF1 showed high sequence identity with HSF in *D. melanogaster*, *Apis cerana*, *Bombyx mori* and *Nilaparvata lugens* (Fig. 1A). A phylogenetic tree was generated using the amino acid sequences of 15 HSF family members in orders Lepidoptera, Diptera, Coleoptera and Hemiptera (Table S1). *Bt*HSF1 grouped in a well-supported cluster with other members of the Hemiptera and was well-separated from insects in other orders (Fig. 2).

### Bthsf1 expression during temperature stress

**Figure 3:**
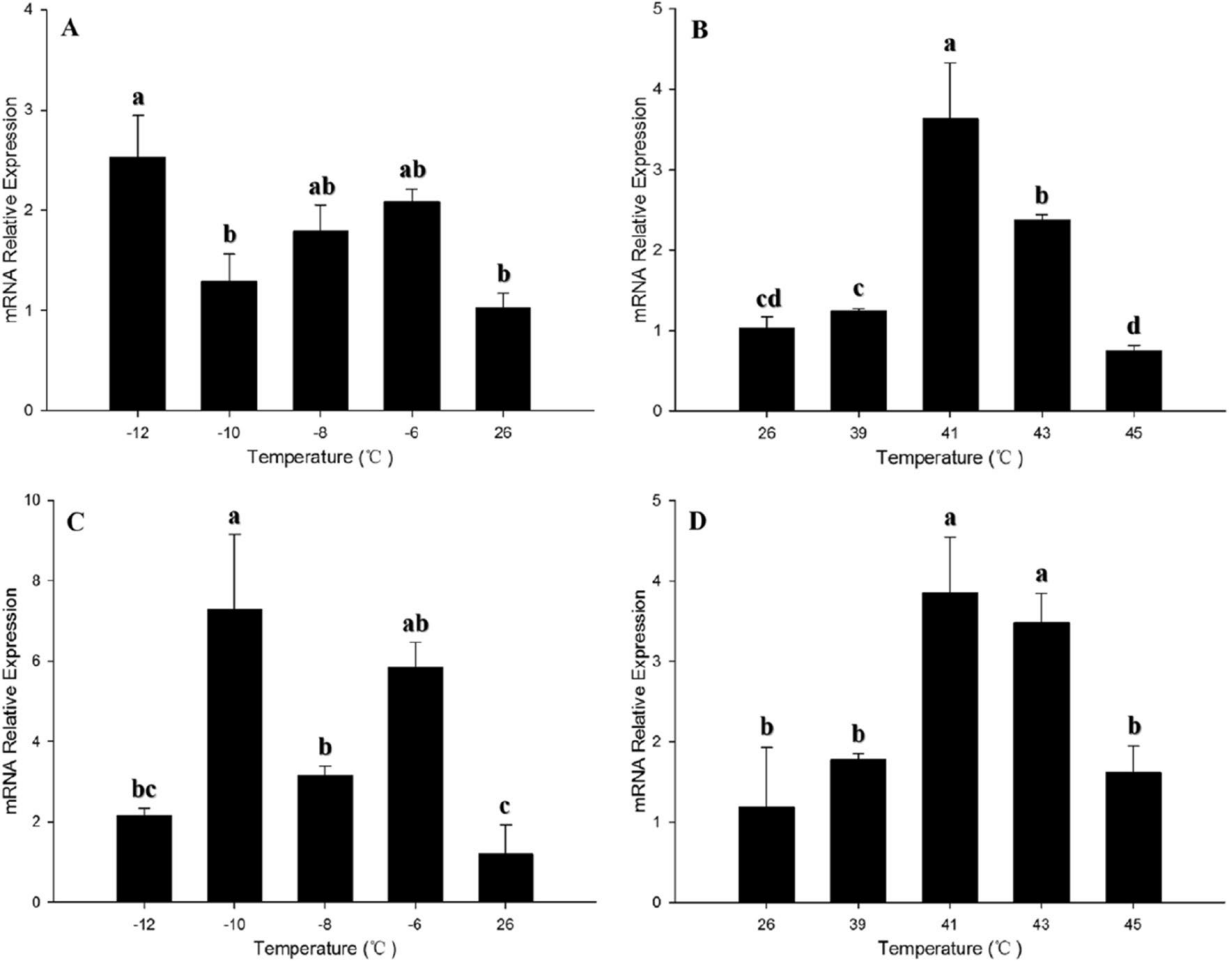
Relative expression levels of *Bthsf1* under thermal stress. (**A**) Adults under cold stress, (**B**) Adults under heat stress, (**C**) Pupae under cold stress, (**D**) Pupae under heat stress. Columns labeled with different letters represent significant differences at *P* < 0.05. The data were denoted as mean ± SE.

The expression of *Bthsf1* was evaluated in response to temperature stress by qRT-PCR. The relative mRNA levels of *Bthsf1* were compared at − 12, − 10, − 8, − 6, 26, 39, 41, 43, and 45 °C for 1 h. *Bthsf1* expression levels were significantly increased at − 12 °C (but not − 10, − 8, and − 6 °C) relative to the control group at 26 °C, which was 2.5-fold great than the control in adults (*F*_*4,15*_ = 5.148, *P* < 0.05). Expression of *Bthsf1* was significantly up-regulated at − 10, − 8, and − 6 °C (but not 12 °C) in pupae, which was highest at − 10 °C and was 7.28-fold greater than the control (*F*_*4,11*_ = 7.645, *P* < 0.05) (Fig. 3A, C).

Compared with the control group (26 °C), expression of *Bthsf1* was significantly up-regulated at 41 °C and 43 °C (but not 39 and 45 °C) in adults and pupae (Adults: *F*_*4,14*_ = 20.324, *P* < 0.05; Pupae: *F*_*4,12*_ = 6.618, *P* < 0.05). *Bthsf1* expression levels were highest at 41 °C, which were 3.6-fold and 3.8-fold greater than the control, respectively (Fig. 3B, D).

### *Bthsf1* expression at different duration of temperature stress

**Figure 4:**
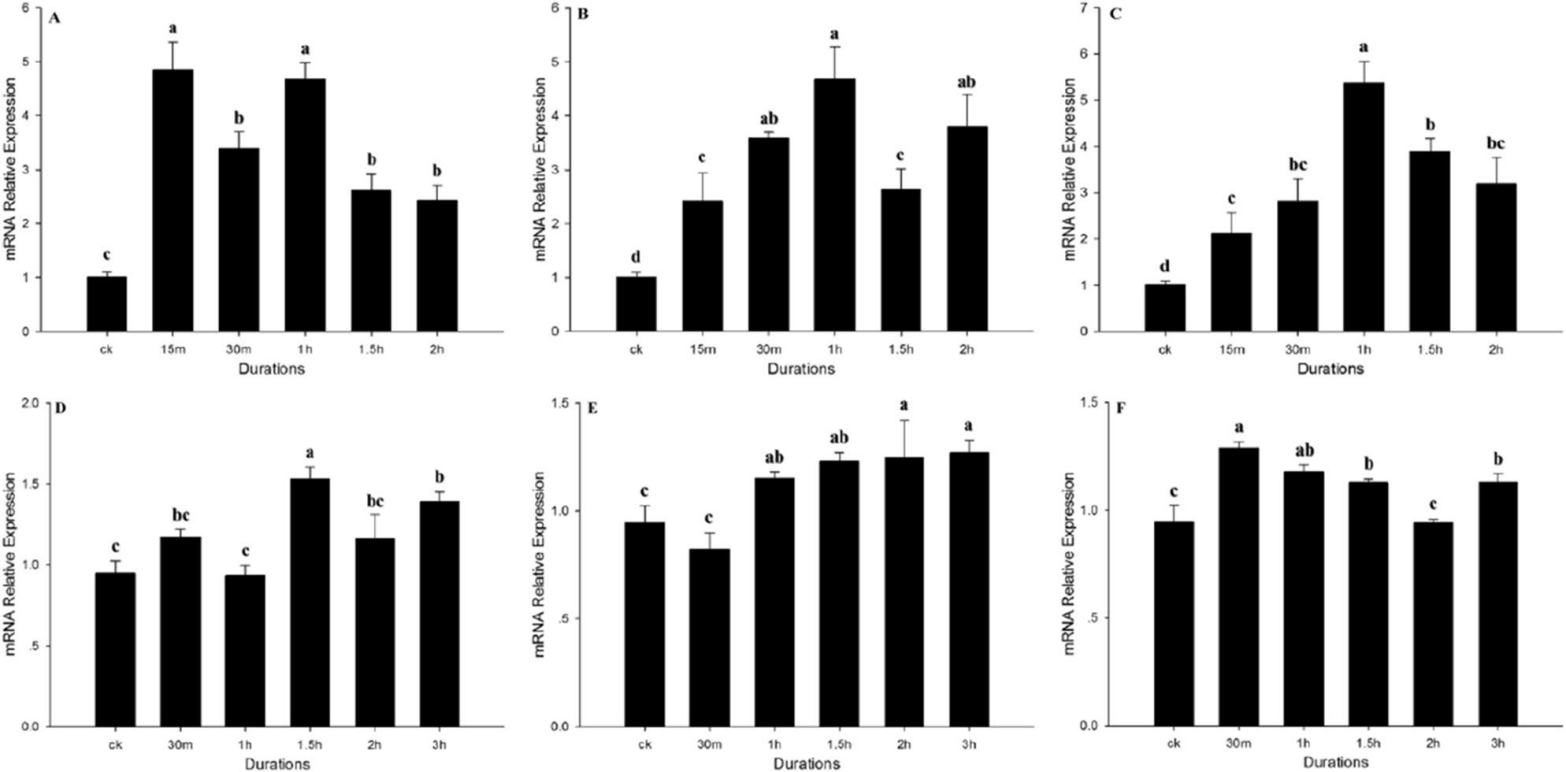
Relative expression levels of *Bthsf* under different duration at heat and cold stress. (**A**) 31 °C, (**B**) 37 °C, (**C**) 43 °C, (**D**) − 10 °C, (**E**) − 4 °C, (**F**) 2 °C. Columns labeled with different letters represent significant differences at *P* < 0.05. The data were denoted as mean ± SE.

qRT-PCR was used to analyze expression of *Bthsf1* during different duration of temperature stress. In this part, 31 °C, 37 °C and 43 °C were selected as high temperatures and the duration of exposure at each temperature was 15 min, 30 min, 1 h, 1.5 h and 2 h. *Bthsf1* expression levels showed different patterns at the three temperatures. At 31 °C, expression levels in the 15 min and 1 h exposure period were 4.9- fold and 4.7- fold greater than the control, respectively (*F*_*5,17*_ = 19.282, *P* < 0.05). At 37 °C and 43 °C, expression levels were highest for the 1 h exposure period, where expression was 4.7- fold and 5.4-fold greater than the control, respectively (*F*_*5,17*_ = 16.166, *P* < 0.05; *F*_*5,15*_ = 15.266, *P* < 0.05) (Fig. 4A, B, C).

Low temperature treatments included exposure to − 10 °C, − 4 °C and 2 °C for 30 min, 1 h, 1.5 h, 2 h and 3 h. Expression levels of *Bthsf1* were significantly increased after exposure to cold stress relative to the control group (ck, 26 °C) (− 10 °C: *F*_*5,17*_ = 7.825, *P* < 0.05;− 4 °C: *F*_*5,17*_ = 4.356, *P* < 0.05; 2 °C: *F*_*5,17*_ = 11.198, *P* < 0.05). However, the multiple of up-regulation is low, and the multiples of up-regulation under different duration treatments are relatively average. It was only found that the expression level of *Bthsf1* was highest at − 10 °C for 1.5 h, at −  °C for 2 h or 3 h and at 2 °C for 30 min, where expression was 1.53-, 1.26- and 1.29-fold greater than the control, respectively (Fig. 4D, E, F).

### Expression of *Bthsf1* and *Bthsps* in RNAi experiments

**Figure 5:**
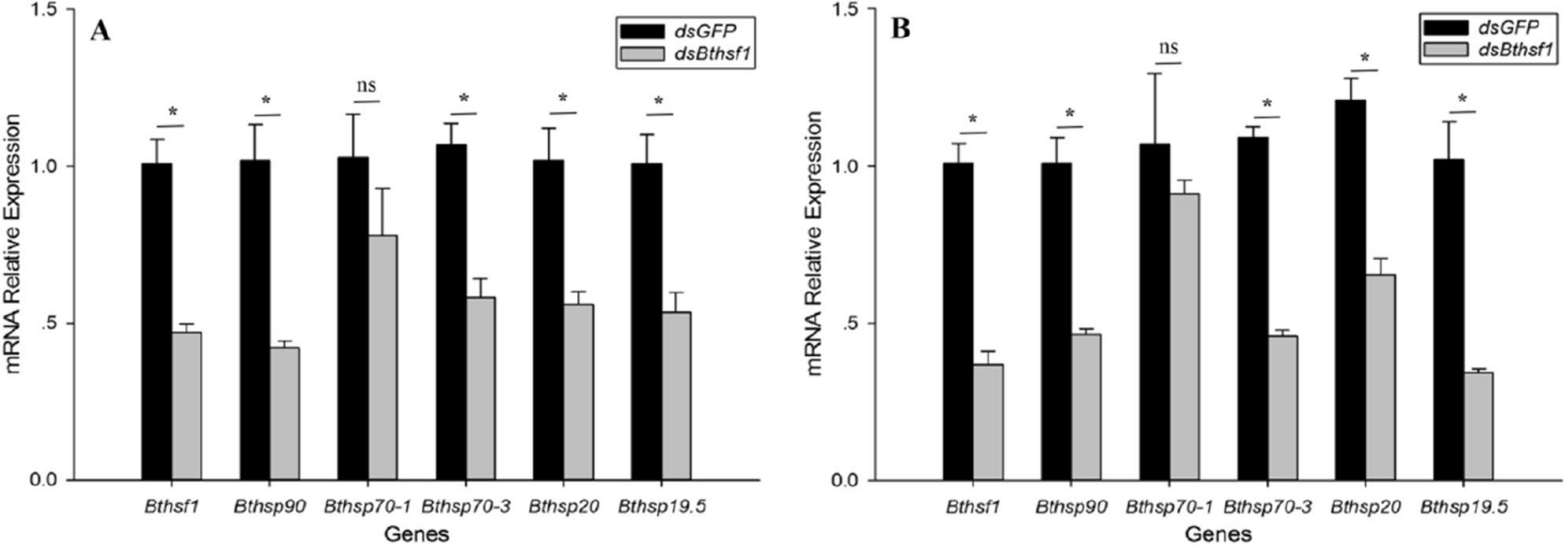
The expression of *hsf1* and *hsps* in *B. tabaci* after oral delivery of *dsBthsf1* and *dsGFP*. (**A**)  − 6 °C, (**B**) 41 °C. Asterisks represent significant differences between *dsGFP* and *dsBthsf1*-treated insects; ns indicates no significant difference.

qRT-PCR analysis showed that mRNA levels of *Bthsf1* were substantially lower when whitefly adults exposured to 41 °C (*t* = 8.456, *P* < 0.05) and − 6 °C (*t* = 6.226, *P* < 0.05) for 1 h (Fig. 5A, B) after whitefly were fed with *dsBthsf1* for 1 day.

The expression levels of *Bthsp90* (HM013710), *Bthsp70-1* (HM013709), *Bthsp70-3* (MK905884), *Bthsp20* (HM013708), and *BtHsp19.5* (MF114301) were evaluated after RNAi and thermal stress. When *B. tabaci* adults were fed with *dsBthsf1* for 1 day, the expression levels of *Bthsp90*, *Bthsp70-3*, *Bthsp20*, and *Bthsp19.5* were significantly down-regulated at − 6 °C relative to the *dsGFP* control (*Bthsp90*: *t* = 5.127, *P* < 0.05; *Bthsp70-3*: *t* = 5.491, *P* < 0.05; *Bthsp20*: *t* = 4.159, *P* < 0.05; *Bthsp19.5*: *t* = 4.334, *P* < 0.05) (Fig. 5A). The same four *Bthsps* were also down-regulated in response to 41 °C (*Bthsp90*: *t* = 6.705, *P* < 0.05; *Bthsp70-3*: *t* = 15.608, *P* < 0.05; *Bthsp20*: *t* = 6.318, *P* < 0.05; *Bthsp19.5*: *t* = 5.593, *P* < 0.05) (Fig. 5B). Interestingly, *Bthsp70-1* was not significantly down-regulated after feeding with *dsBthsf1* at either temperature stress ( − 6 °C: *t* = 2.334, *P* = 0.058; 41 °C: *t* = 0.695, *P* = 0.513).

### Mortality of *B. tabaci* after RNAi

**Figure 6:**
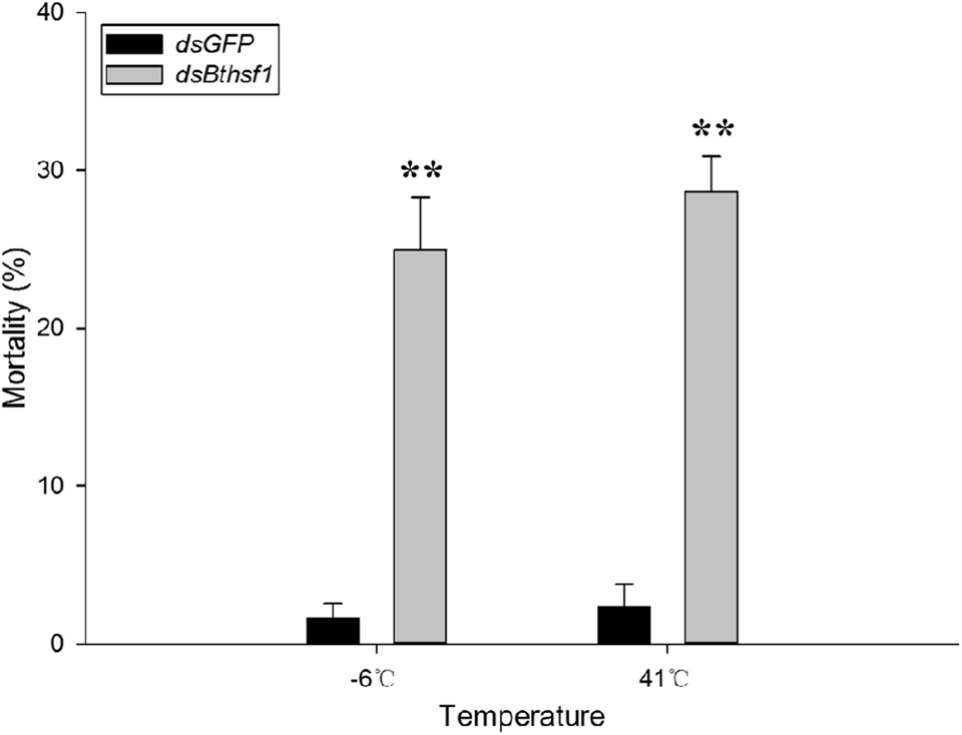
Effects of thermal treatments on the mortality of dsRNA-ingested *B. tabaci*. Mortality of *B. tabaci* was determined after thermal treatments for 1 h. Asterisks represent significant differences between *dsGFP* and *dsBthsf1*.

Mortality was measured after feeding *B. tabaci* with *dsBthsf1* or *dsGFP* and then exposing adults to thermal stress. Mortality of *B. tabaci* fed with *dsBthsf1* was 23% and 26% more than the *dsGFP* control at − 6 °C (*t* = 9.690, *P* < 0.05) and 41 °C (*t* = 6.759, *P* < 0.05), respectively (Fig. 6).

## Discussion

A variety of internal and external stimuli can activate HSF, including heat shock. There are three key steps in HSF function in heat stress including the following: polymerization of HSF from monomer to trimer; recognition and binding of HSF to the HSE in *hsp* promoter regions; and transcriptional activation of the *hsps*^37^. Therefore, it is important to study how HSF regulates the expression of genes encoding HSPs when insects undergo thermal stress.

In this study, we cloned and identified the *Bthsf1* in *B. tabaci* MED cryptic species. The deduced *Bt*HSF1 contains the conserved motif (DNA-binding domain, DBD) of the HSF family. The predicted amino acid sequence of *Bthsf1* shows considerable sequence similarity with HSF orthologues in *D. melanogaster*, *B. mori*, *A. cerana* and *N. lugens*. Phylogenetic analysis revealed that *Bt*HSF1 resides within a phylogenetic group that includes HSF in other Hemiptera insects, including *Nl*HSF in the brown planthopper (*N. lugens*), *Ag*HSF in the cotton aphid (*Aphis gossypii*) and *Dn*HSF in the Russian wheat aphid (*Diuraphis noxia*). The phylogenetic conservation of HSF within the Hemiptera indicates that *Bt*HSF1 could be potentially useful in taxonomic studies. By modeling the 3D structure of *Bt*HSF1, we found that it has a high degree of similarity at the conservative sequence (DBD) structure with *Drosophila melanogaster*, indicating that the *Bt*HSF we obtained has the typical characteristics of the insect HSF1 family. Combined with multiple sequence comparison analysis results, it also shows that HSF1 family genes in insects have a strong conservativeness at the characteristic sequence (DBD) structure.

Under normal conditions, HSF exists as an inactive monomer in the cytoplasm and is bound to HSPs^15^. When cells are subjected to thermal stress, the internal environment shifts, which relieves the inhibition of HSF activity. Interestingly, there are relatively few studies documenting *hsf* expression patterns in insects during temperature stress. Our results show that *Bthsf1* can be significantly activated and expressed constitutively by high and low thermal stress, It shows that this transcription factor can interact with the HSEs when the whitefly resists external temperature stress, and promote the expression of HSPs^33,35,36^. In *D. melanogaster*, the transcription of *Dmhsf, Dmhsfb* and *Dmhsfd* were upregulated during temperature stress^17,38^, and genes encoding HSF in *Mamestra brassicae* and *Agasicles hygrophila* was also induced by thermal stress^21,39^, However, it is important to note that there obvious differences in *hsf* expression among insects; for example, *Cchsf* encoding HSF in *Cotesia chilonis* was induced by low but not high temperature stress^40^.

Several studies have shown that the duration of temperature stress impacts the growth and development of organisms^41–43^. In this study, we analyzed *Bthsf1* expression during different duration of temperature stress. *Bthsf1* had obvious peak expression levels during high temperature stress; e.g. prominent peaks at 15 min (31  °C) and 1 h (31  °C, 37  °C, 43 °C). However, these spikes in *Bthsf1* transcript levels were not observed during low temperature stress. Our results indicate that *B. tabaci* is conditioned by heat stress to activate HSF1 and promote *hsp* expression. Furthermore, our findings help explain why the fold increases in *Bthsps* transcription during high temperatures are so much higher than *hsp* expression levels during cold temperatures^33,36^. During prolonged periods of heat stress, *Bthsf1* is gradually down-regulated; the protracted accumulation of HSPs becomes deleterious to the cell, which leads to the repression of HSF by HSP70 and other molecular chaperones^44–46^. Our results reveal the importance of studying the expression of *hsf* and *hsp* concurrently during thermal stress.

The feeding method of dsRNA delivery has been widely and successfully used to study gene function in hemipteran insects^47–52^. When *B. tabaci* was supplied with *dsBthsf1* for 1 day, *Bthsf1* expression was significantly downregulated after exposure to  − 6  °C and 41  °C, and mortality increased relative to the *dsGFP* control. The contribution of HSF to *hsp* expression, fecundity and survival during adverse conditions has been studied in other organisms. For example, in *Artemia franciscana*, *hsf1* knockdown decreased *hsp* expression in diapausing embryos^53^, and RNAi-mediated suppression of *hsf* in *Haliotis diversicolor* downregulated several *hsps*^54^. In *A. hygrophila*, microinjection of *dsAhHsf* into newly-emerging adults reduced the expression of two different *hsps* and decreased egg production and survival^39^. In our study, RNAi with *dsBthsf1* resulted in a significant down-regulation of *Bthsp90*, *Bthsp70-3*, *Bthsp20*, and *Bthsp19.5* at − 6  °C and 41  °C, indicating that *Bthsf1* is involved in the regulation of multiple HSP genes in *B. tabaci*. In addition, we also found that the expression of *Bthsp70-1* did not decrease as knockdown of *Bthsf1*, indicating that *Bthsf1* may not be the most important regulatory path for *Bthsp70-1*, and there may be other ways to regulate the expression of the gene. Collectively these findings indicate that *Bthsf1* can regulate the expression of some but not all *hsps*, and further studies are warranted to confirm *Bt*HSF1 interactions and regulatory functions.

## Materials and methods

### Insects

*B. tabaci* were reared on tomato (The tomato seeds involved in this study are in line with the national seed quality standards in China, the implementation standard number is GB16715.3–2010, and the seed production and operation license number is: D (Jicangqing) Nongzhongxuzi (2016) No.0006, Xingyun Vegetable Breeding Center, Hebei, China) in controlled temperature chambers plants as described^36^. Identification of the *B. tabaci* MED cryptic species was determined using the mitochondrial cytochrome oxidase I (mtCOI) gene as described previously^55^.

### Isolation of RNA, cloning and RACE

Total RNA was isolated from *B. tabaci* pupae and adults as described previously and stored at − 80 °C until needed^36^. cDNA was synthesized using an oligo(dT)_18_ primer (TaKaRa), and full-length cDNAs encoding HSF1 were obtained by 5′- and 3′-RACE (SMART RACE, Clontech) using the primers listed in Table 1. HSF sequences were confirmed by 5′ RACE.

**Table 1:** Primer sequences used in the cDNA cloning, Quantitative real-time PCR and dsRNA synthesis.

Genes	Primer sequence (5′→3′)		
RACE			
*Hsf1*			
F	TCACGGAAGTAGGGACGAATGT		
R	CACTGTGTTGTCTGGCATACTG		
5′	AGAGACATTCGTCCCTACTTCCGT		
3′	GTTACTGGTTCTCTACCTCTGGAT		
qRT-PCR		R^2^	E (%)
*Hsf1*		0.991	96.5
F	TCTGGTCCATCCCACACG		
R	CTGCCGCTGTTGTAGTGC		
*Hsp90*		0.985	98.4
F	ACAACTTGGGAACAATCGCC		
R	ATAGAGATGTCAGCACCAGC		
*Hsp70-1*		0.995	99.0
F	GAAGAACTTTGCTCTGACCT		
R	TATCCATTTTCGCATCAGCC		
*Hsp70-3*		0.994	100.5
F	CCTACGGATTGGATAAGAACCTG		
R	GCAGTTGCTCGCACTTCAAATAG		
*Hsp20*		0.990	106.1
F	ACCAACCCACTCTCCGCAC		
R	ACTCCACTCTGCTGGGCTG		
*Hsp19.5*		0.988	99.8
F	TTCACCCGCCGTTACCTTCTGC		
R	TGCCTTTGGTGGAGCCTGGATG		
*EF-1α*		1	95.3
F	TAGCCTTGTGCCAATTTCCG		
R	CCTTCAGCATTACCGTCC		
*RPL29*		1	96.6
F	TCGGAAAATTACCGTGAG		
R	GAACTTGTGATCTACTCCTCTCGTG		
dsRNA synthesis			
*dsBthsf1 *-F	TAATACGACTCACTATAGGG		
	CACATCAAAACTTAGAAATAATT		
*dsBthsf1 *-R	TAATACGACTCACTATAGGG		
	AGTCTTCTTCCATCTTTTGACTT		
*dsGFP *-F	TAATACGACTCACTATAGGG		
	CCTCGTGACCACCCTGACCTAC		
*dsGFP *-R	TAATACGACTCACTATAGGG		
	CACCTTGATGCCGTTCTTCTGC		

### Isolation and characterization of *Bthsf1*

The fragment of HSF1 was isolated and identified based on analysis of the published transcriptome data^56^. The primers used for amplifying fragment are provided in Table 1. PCR products were purified, cloned, and sequenced as described^36^.

Established methods were used for identifying ORFs and aligning amino acid sequences^36,57^. *Bthsf1* sequences were analyzed with tools available at the ExPASy Molecular Biology Server (https://www.expasy.org/) including Compute pI/MW, BLAST, and Translate. Phylogenetic analyses were conducted as described previously^36,58^. The three-dimensional (3D) structure of the DBD domain was predicted by the SWISS-MODEL website (https://swissmodel.expasy.org/) using the *Drosophila melanogaster* DBD domain (SMTL ID: 1hkt.1) as a template.

### Synthesis of dsRNA

Full-length *B. tabaci* HSF1 gene was identified using the online website (http://sidirect2.rnai.jp/); the regions for RNA silencing were determined, and primers for RNAi were designed. Sense and antisense primers included a T7 promoter sequence (TAATACGACTCACTATAGGG) at the 5′ ends to catalyze transcription from both cDNA strands (Table 1). dsRNA specific to the gene encoding green fluorescence protein (*dsGFP*) was used as a control (Table 1). PCR products were cloned in pGEM-T easy (Promega, Madison, WI, USA) and resulting constructs were used as template DNA in subsequent amplifications. The PCR product was used for preparation of double-stranded RNA (dsRNA) using the MEGAscript® RNAi kit according to the manufacturer's instructions (Thermo, Waltham, MA, USA)^59^. The quality of dsRNA was evaluated by spectrophotometry and gel electrophoresis and then diluted into 30% (w/v) sucrose for use in experiments.

### Oral ingestion of dsRNA

Feeding chambers for delivering dsRNA were constructed as described previously^60^ with minor modifications. Two pieces of Parafilm membrane (2 × 2 cm^2^) were stretched out by hand until they were each twofold their original length. A tube was sealed with 2 layers of membrane containing 30% (w/v) sucrose solution (300 μL) between them. One side of the tube was covered with a piece of meshed net to allow aeration. Adult whiteflies (aged less than 12 h) were released into the Parafilm chamber before covering it with a meshed net. A Parafilm sandwich was positioned into the top of the tube and the tube was incubated at 25  °C For experiments, various amounts (500 ng/μL) of *dsBthsf1* or *dsGFP* were diluted into 30% (w/v) sucrose solution. Experiments were conducted four times under identical conditions.

### Temperature exposure

*B. tabaci* adults and pupae were collected, placed in glass tubes, and exposed to each of the following temperatures for 1 h: − 12,− 10,− 8,− 6, 39, 41, 43, and 45 °C. Adults and pupae that were maintained at room temperature (26  °C) were used as controls. Treated adults and pupae were allowed to recover at 26 °C for 1 h and were then frozen in liquid nitrogen and stored at − 80 °C (*N* = 4).

In experiments with different duration of temperature, *B. tabaci* adults (*n* = 60) were exposed to high temperatures (31, 37 and 43 °C) for 15 min, 30 min, 1 h, 1.5 h, and 2 h and low temperatures ( − 10, − 4 and 2 °C) for 30 min, 1 h, 1.5 h, 2 h and 3 h. Insects were then allowed to recover at 26 °C for the same duration as the temperature treatment (*N* = 4). Adults and pupae maintained at room temperature (26 °C) were used as controls.

For RNAi, newly emerged *B. tabaci* adults were supplied with *dsBthsf* or *dsGFP* for 1 day, exposed to -6 and 41 °C for 1 h, and then allowed to recover at 26 °C for 1 h. The mortality of *B. tabaci* was checked after temperature stress, and the surviving *B. tabaci* were frozen in liquid nitrogen and stored at − 80 °C. Each treatment included four biological replications.

### Quantitative real-time PCR

The cDNA template was transcribed from RNA with the HiScript III RT SuperMix for qPCR (Vazyme, Nanjing, China) as recommended, and primers were designed with Primer 5.0 software (Table 1). Quantitative real-time PCR (qRT-PCR) was performed in 20 μL total reaction volumes comprised of 10 μL of 2 × ChamQ Universal SYBR qPCR Master Mix (Vazyme, Nanjing, China), 1 μL of each gene specific primer (Table 1), and 2 μL of cDNA templates. It was carried out that reactions on a CFX-Connect real-time PCR system (Bio-Rad, Berkeley, CA, USA) using the following conditions: 3 min at 95 °C, 40 cycles of denaturation at 95 °C for 30 s, and annealing (30 s) at 60 °C for each gene. And gene expression was calculated using the 2^−ΔΔCt^ method and normalized to the abundance Elongation factor 1 alpha (*EF-1α*) and 60S ribosomal protein L29 (*RPL29*)^61^.

### Data analysis

One-way ANOVA, followed by Tukey’s and Duncan’s multiple comparison, was used to detect significant differences among temperatures using SPSS v. 16.0^62^. For ANOVA, data were transformed for homogeneity of variances, and differences were considered statistically significant when *P* < 0.05.

For RNAi, the relative abundance of target genes and survival rates were compared to the *dsGFP* control. Student’s t-test was used to compare differences in gene expression and mortality with SPSS v. 16.0, and differences were considered significant at *P* < 0.05.

## Supplementary Information

Supplementary Information 1.

Supplementary Information 2.

## Data Availability

The datasets used and/or analysed during the current study available from the corresponding author on reasonable request.
