# Supplementary material for: Knockdown of heat shock transcription factor 1 decreases temperature stress tolerance in Bemisia tabaci MED
Source: Sci Rep. 2022 Sep 26;12:16059. doi: 10.1038/s41598-022-19788-z (PMC9512819; doi:10.1038/s41598-022-19788-z)
Supplement: Supplementary file 1 — Supplementary Information 1. [file 41598_2022_19788_MOESM1_ESM.docx]

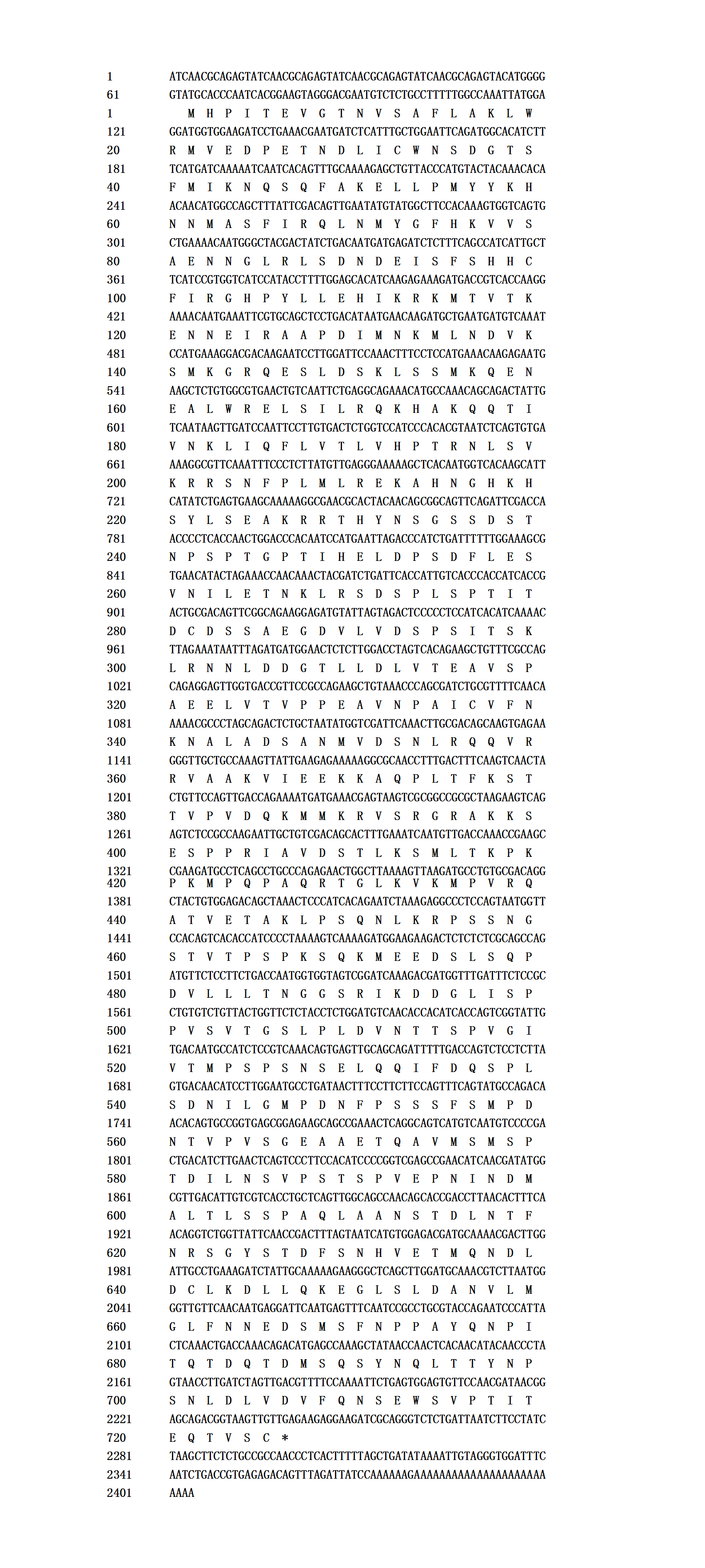


**Figure S1.** Nucleotide and deduced amino acid sequences of heat shock transcription factor 1 gene in *Bemisia tabaci* MED.
