# Supplementary material for: Knockdown of heat shock transcription factor 1 decreases temperature stress tolerance in Bemisia tabaci MED
Source: Sci Rep. 2022 Sep 26;12:16059. doi: 10.1038/s41598-022-19788-z (PMC9512819; doi:10.1038/s41598-022-19788-z)
Supplement: Supplementary file 2 — Supplementary Information 2. [file 41598_2022_19788_MOESM2_ESM.docx]

Table S1. Inferred amino acid sequence identities of HSF in *Bemisia tabaci* with its homologs from other insects.

| Species | | Type | GenBank accession number |
| --- | --- | --- | --- |
| Hemiptera | *Bemisia tabaci* | *Bthsf1* | MW478139 |
|  | *Diuraphis noxia* | *Dnhsf* | XP_015372325 |
|  | *Nilaparvata lugens* | *Nlhsf* | XP_022189904 |
|  | *Aphis gossypii* | *Aghsf* | XP_027847816 |
| Coleoptera | *Anoplophora glabripennis* | *Aghsf1* | XP_018573159 |
|  | *Tribolium castaneum* | *Tchsf* | XP_008199012 |
| Diptera | *Bactrocera dorsalis* | *Bdhsf* | XP_029406933 |
|  | *Drosophila melanogaster* | *Dmhsf* | AAA28642 |
|  | *Drosophila simulans* | *Dshsf* | XP_016028338 |
|  | *Musca domestica* | *Mdhsf* | XP_019890613 |
| Lepidoptera | *Bombyx mori* | *Bmhsf* | BAK26393 |
|  | *Galleria mellonella* | *Gmhsf* | XP_026759219 |
|  | *Helicoverpa armigera* | *Hahsf* | ANW82404 |
|  | *Mamestra brassicae* | *Mbhsf* | BAG07219 |
|  | *Spodoptera exigua* | *Sehsf* | AYI99246 |
|  | *Spodoptera frugiperda* | *Sfhsf* | QGA73371 |
